# Complete identity and expression of StfZ, the *cis*-antisense RNA to the mRNA of the cell division gene *ftsZ*, in *Escherichia coli*

**DOI:** 10.3389/fmicb.2022.920117

**Published:** 2022-10-19

**Authors:** Deepak Anand, Kishor Jakkala, Rashmi Ravindran Nair, Deepti Sharan, Atul Pradhan, Nagaraja Mukkayyan, Parthasarathi Ajitkumar

**Affiliations:** ^1^Department of Microbiology and Cell Biology, Indian Institute of Science, Bengaluru, India; ^2^Department of Biology, Lund University, Lund, Sweden; ^3^Department of Microbiology and Immunology, Emory University School of Medicine, Atlanta, GA, United States; ^4^Department of Microbiology, University of Alabama at Birmingham, Birmingham, AL, United States; ^5^Department of Microbiology, The University of Chicago, Chicago, IL, United States; ^6^Department of Medicine, Renaissance School of Medicine, Stony Brook University, Stony Brook, NY, United States; ^7^Department of Microbial Pathogenesis, University of Maryland, Baltimore, MD, United States

**Keywords:** *Escherichia coli*, StfZ *cis*-antisense RNA, *ftsZ* mRNA, FtsZ level, cell division

## Abstract

Bacteria regulate FtsZ protein levels through transcriptional and translational mechanisms for proper cell division. A *cis*-antisense RNA, StfZ, produced from the *ftsA-ftsZ* intergenic region, was proposed to regulate FtsZ level in *Escherichia coli*. However, its structural identity remained unknown. In this study, we determined the complete sequence of StfZ and identified the isoforms and its promoters. We find that under native physiological conditions, StfZ is expressed at a 1:6 ratio of StfZ:*ftsZ* mRNA at all growth phases from three promoters as three isoforms of 366, 474, and 552 nt RNAs. Overexpression of StfZ reduces FtsZ protein level, increases cell length, and blocks cell division without affecting the *fts*Z mRNA stability. We did not find differential expression of StfZ under the stress conditions of heat shock, cold shock, or oxidative stress, or at any growth phase. These data indicated that the *cis*-encoded StfZ antisense RNA to *ftsZ* mRNA may be involved in the fine tuning of *ftsZ* mRNA levels available for translation as per the growth-phase-specific requirement at all phases of growth and cell division.

## Introduction

FtsZ is an essential protein for cell division and cytoskeletal integrity in most bacteria (Dai and Lutkenhaus, 1991; Pla et al., 1991; Löwe et al., 2004; Shih and Rothfield, 2006). In *Escherichia coli*, the ratio between FtsZ and FtsA molecules (5:1) (Rueda et al., 2003) is important for proper cell division (Ward and Lutkenhaus, 1985; Dai and Lutkenhaus, 1992; Dewar et al., 1992). It was shown that a 2- to 7-fold increase in FtsZ level results in mini-cell formation, due to additional division events, whereas further higher levels or lower than normal levels cause filamentation due to imbalance in the FtsZ: FtsA stoichiometry (Bi and Lutkenhaus, 1990; Wang and Gayda, 1990). Thus, regulation of FtsZ level is crucial for proper cell division. The regulation of *ftsZ* occurs at the transcriptional and translational levels (Aldea et al., 1990; Cam et al., 1996; Flärdh et al., 1997; Flynn et al., 2003; Tamura et al., 2006; Camberg et al., 2009).

Trans-acting small non-coding antisense RNAs (asRNAs), which are usually encoded in the intergenic regions on the chromosome, control translation, or degradation of their target mRNAs. Generally, each trans-acting non-coding asRNA has multiple target mRNAs and binds near their ribosomal binding site (Saberi et al., 2016). A structural change in the non-coding RNA occurs through binding to small metabolites (riboswitches) or through a change of temperature (thermoregulators) or pH (pH sensors) (Saberi et al., 2016). In both cases, elevated temperature caused the phenotypic effects of asRNAs. One such asRNA, DicF, against *ftsZ* has been found to influence FtsZ protein level in certain strains of *Escherichia coli* (Tétart and Bouché, 1992). Besides DicF, Dewar and Donachie had proposed the expression of StfZ *cis*-asRNA, from the 60-bp spacer sequence and extending into the 5′ portion of *ftsZ*, which blocks cell division when placed in a high copy number plasmid at 42°C (Dewar and Donachie, 1993). However, StfZ was not studied further for its expression levels or promoters controlling its expression. Since regulation of the principal cell division gene *ftsZ* is crucial for cell division control, it is important to elucidate the mode of action and physiological role of StfZ *cis*-asRNA in *E. coli* cell division.

This study establishes the complete sequence of StfZ, its growth-phase-dependent expression, the stoichiometry of its expression with *ftsZ* mRNA, and its role in cell division. We showed that StfZ RNA has three isoforms transcribed from three promoters. The three isoforms are expressed at relatively similar levels. Further, we investigated its influence on FtsZ level and thereby on cell division. The generation of a knockout or deletion mutant of the *stfZ* was not possible as the StfZ sequence is overlapping with the sequence of the essential cell division gene, *ftsZ*. Nevertheless, the observations reported in this study show the expression of natural antisense RNA isoforms of StfZ as a novel factor that affects *ftsZ* mRNA level and thereby FtsZ protein level, as per the demand for growth and cell division in *E. coli*.

## Materials and methods

### Bacterial strains, plasmids, and growth

Bacterial strains and plasmids are listed in Supplementary Tables 1, 2, respectively. All the strains were cultured in Luria-Bertani broth or agar for growth. Strains with plasmids were selected on ampicillin (100 μg/ml) or kanamycin (25 μg/ml). For StfZ, induction cultures were grown at 30°C and shifted to 37 or 42°C as per the experiment. All the cultures were balanced for OD and volume for the induction and stress experiments.

### cDNA preparation

RNA was isolated using hot phenol method (Wecker, 1959; Roy et al., 2004). In brief, cells were lysed in lysis buffer (Supplementary Table 3). The aqueous phase of RNA was extracted with hot phenol (65°C, pH 5.2) followed by phenol: chloroform and chloroform extractions. RNA was precipitated and dissolved in RNase-free water. RNA preparations were treated with DNase-I which was verified using PCR for a 16S rRNA gene (Condon et al., 1995). RNA samples were loaded on 1% formaldehyde agarose gel to check the quality of RNA. cDNA was prepared using 5 μg total RNA with RevertAid-Premium Reverse Transcriptase kit (Fermentas). For each reaction, 20 pmoles of gene-specific reverse primer were added and annealed at 55°C for 10 min, followed by the addition of reverse transcriptase for extension at 55°C for 60 min. The reaction was stopped by incubating at 85°C for 10 min. The cDNA preparation was used for RT-PCR and quantitative PCR.

### RT-PCR and real-time polymerase chain reaction

RevertAid-Premium Reverse-Transcriptase kit and Evagreen real-time PCR master mix (GBiosciences) were used for RT-PCR and real-time PCR, respectively (Wang et al., 2006). Primers ODA-01 and ODA-02 for StfZ, ODA-03 and ODA–04 for 16S rRNA, ODA–05 and ODA–06 for *ftsZ*, ODA–09 and ODA–10 for *mutgfp*, ODA–11 with ODA–12 for *ftsA*, ODA–40 and ODA–41 for *csp*A, ODA–42 and ODA–43 for *rpo*H, and ODA–44 and ODA–45 for *kat*G (Supplementary Table 4) were used. StfZ ODA–46 and ODA–47 for region “a,” ODA–48- and ODA–49 for region “b,” and ODA–50 and ODA–51 for region “c” were used for real-time PCR for the differential amounts of the three isoforms of StfZ RNA (Supplementary Table 4). Reactions were performed as per the described protocols. The cDNA of 16S rRNA was used as the normalisation control (Condon et al., 1995). Real-time PCR was performed in Applied Biosystems-ViiA7. The 2^–ΔΔ*Ct*^ method was used for quantitation (Livak and Schmittgen, 2001; Giangrossi et al., 2010). The fold changes of expression were presented as expressions relative to the control sample.

### Stress induction

We tested heat-shock (46°C) (Grossman et al., 1984), cold-shock (16°C) (Etchegaray et al., 1996), and oxidative stress (5 mM H_2_O_2_) (Schellhorn, 1995) conditions in WT cells from mid-log (OD 0.6). Cells were grown at 30°C to mid-log (OD 0.6) and then split into four sets. Each set was then exposed to 30 min of stress condition or untreated condition. Real-time PCR was performed for StfZ and *fts*Z mRNA. Corresponding known gene expression was also included for confirmation of the occurrence of stress responses. For heat shock response, *rpo*H expression; for cold shock, *csp*A expression and oxidative stress *kat*G expression were measured by real-time PCR (Grossman et al., 1984; Schellhorn, 1995; Etchegaray et al., 1996). For the gene expression control, expression level from the uninduced culture was used.

### Primer extension assay

Primer extension assay was performed using 30 μg of total RNA isolated from *E. coli* K12 cells (Blattner et al., 1997) of 0.3 OD_600nm_ (OD). Primers, ODA-07 and ODA-08, were radiolabelled with [γ-^32^P]-ATP using T4-polynucleotide kinase kit (Fermentas). Labelled primers were purified with Sephadex G-50 column, annealed to RNA, and extended at 55°C with 200 U of RevertAid-Premium Reverse-transcriptase for 60 min. Primer extension products were denatured at 95°C and fractionated on 8% polyacrylamide gel containing 7 M urea. A parallel manual sequencing reaction was performed using CycleReader™ DNA Sequencing Kit (Fermentas) and loaded in the lane next to the PE reaction. The PCR product template for sequencing was generated using primer ODA-13 in combination with ODA-08 or ODA-07 (Supplementary Table 4). Autoradiography was performed using a phosphorimager after 24 h of exposure to the sample.

### Molecular cloning

The oligonucleotide primers used in cloning are listed in Supplementary Table 4 and the plasmids are listed in Supplementary Table 2.

#### pDA1

Linear 3′ RACE product was treated with T4 polynucleotide kinase and then ligated to *Eco*RV site in pBS(KS) (Alting-Mees and Short, 1989).

#### pDA2, pDA3, pDA4, pDA5, pDA6, and pDA7

StfZ promoters were cloned using oligonucleotide annealing method (Arumugam et al., 2012). For pDA2, ODA-16 and ODA–17, for pDA4, ODA-18 and ODA-19, and for pDA6, ODA–20 and ODA–21 oligos were annealed and cloned at *Kpn*I and *Bam*HI site in pFPV27 (Valdivia and Falkow, 1996). Similarly in case of −10 element deletion, for pDA3, ODA–22 and ODA–23, for pDA5, ODA–24 and ODA–25 for pDA7 ODA–26 and ODA–27 oligos were used.

#### pDA8

The entire region spanning the promoters P1 to P3 (including the −10 and −35 elements of the respective promoters) was amplified using ODA–28 and ODA–29 (Supplementary Table 4) and cloned between the *Kpn*I and *Bam*HI sites in the promoter probe vector, pFPV27.

#### pDA9

Region of *stfZ* was amplified from *E. coli* genomic DNA using primer ODA–14 and ODA–15. PCR product was digested with *Kpn*I and *Xba*I and ligated to pBS(KS) vector at the same sites.

#### pDA10

Cloning was performed by a reverse PCR on pDA9 using primer ODA–32 and ODA–33. The linear product was self-ligated after polynucleotide kinase treatment.

#### pDA11, pDA12, and pDA13

For substitution mutations of promoters, pDA11, ODA–34, and ODA–35; for pDA12, ODA–36, and ODA–37; and for pDA13, ODA–38, and ODA–39 were annealed and cloned at *Kpn*I and *Bam*HI site in pFPV27.

### Promoter assay

Putative StfZ promoters and its entire-10 region deletion mutation or substitution mutation constructs (Supplementary Table 2) were expressed from JM109 strain (Yanisch-Perron et al., 1985) (Supplementary Table 1). cDNAs for *mutgfp* were synthesised using primer ODA–10 from total RNA isolated from the promoter construct transformants grown to the mid-log phase (0.6 OD). Promoter activity was quantitated using real-time PCR for *mutgfp* mRNA. cDNA was synthesised with ODA–10 oligo, and PCR was performed using the combination of ODA–09 and ODA–10 (Supplementary Table 4).

### 3′ rapid amplification of cDNA ends

Twenty micrograms of total RNA from 0.5 OD_600 nm_ culture was enriched for total mRNA using Ribominus™ Transcriptome Isolation kit (Invitrogen K1550–03). The 5′ phosphorylated ODA–30 oligo (Supplementary Table 4) was ligated to the 3′ ends of ribominus RNA using T4 RNA ligase. The cDNA was synthesised with the complementary oligo ODA–31 (Supplementary Table 4). StfZ-specific cDNA was PCR amplified using ODA–01 and ODA–31 primers, using standard conditions. The PCR product was gel eluted and cloned in pBS(KS). The insert was sequenced.

### Northern hybridisation

RNA probe was generated against the *stfZ* region by *in vitro* transcription from *Kpn*I digested pDA9 plasmid. HiScribe™ T7 High Yield RNA synthesis kit (NEB, Gothenburg, Sweden) was used as per the manufacturer’s protocol with Biotin Labelling RNA Mix (Roche, Solna, Sweden) to obtain the *stfZ* complementary probe. About 100 μg total RNA from PAK02 and PAK12 strains (0.2 OD) were fractionated on 10% polyacrylamide denaturing gel with 7 M urea. RNA was blotted to a positively charged nylon membrane (BrightStar-™Plus, ThermoFischer Scientific, Gothenburg, Sweden). The membrane was subjected to UV cross-linking (1200 μJ/cm^2^ for 20 min). The membrane was blocked in a pre-hybridisation buffer (Supplementary Table 3) at 60°C for 3 h. Pre-hybridisation buffer was replaced with hybridisation buffer containing 5 μg biotin-labelled RNA probe (denatured at 65°C for 5 min and snap-chilled on ice) and incubated overnight at 60°C. The nylon membrane was washed thrice with wash buffer (1x SSC containing 0.1% SDS) for 15 min each, at room temperature. The membrane was then blocked for 15 min and incubated with streptavidin-HRP conjugate (Invitrogen) (1:10,000 dilution) for 15 min. The membrane was washed and developed using Clarity Western ECL Substrate (Bio-Rad, Solna, Sweden).

### Measurement of YFP fluorescence

The culture PAK13 was grown at 30°C and induced at 0.6 OD with either 0.1% arabinose (for *ftsZ-yfp* mRNA) or with 0.1% arabinose and 1 mM IPTG (for StfZ RNA) simultaneously. At 120 min of induction, the cells were placed on a glass slide and imaged for YFP fluorescence. Expression of FtsZ-YFP was measured from the cells and normalised to the area of the cell.

### Immunofluorescence microscopy

Immunofluorescence microscopy was performed as described (Addinall et al., 1996), with a few modifications. The harvested cells were fixed with 0.4% paraformaldehyde and 0.25% glutaraldehyde solution for 10 min at room temperature and 50 min on ice. The cells were washed with 1x PBS (pH 7.4) (Supplementary Table 3), layered over poly-L-lysine (0.1%, w/v) coated multi-well slide, permeabilised with 2 mg/ml of lysozyme (Sigma), blocked with BSA (2% w/v in PBS), followed by incubation with 1:500 dilution of affinity-purified rabbit polyclonal anti-FtsZ antibody overnight at 4°C in a humid chamber. The cells were washed five times with 1x PBS, followed by 60 min incubation with 1:1000 dilution of Cy3 anti-rabbit IgG antibody (0.1 μg/ml; Sigma, Bengaluru, India). The cells were washed again with 1x PBST (Supplementary Table 3) and incubated with 0.5 μg/ml DAPI for 5 min. DAPI was washed off with 1x PBST solution and cells were mounted with 80% glycerol. Images were taken under the Zeiss AxioImager M1 fluorescence microscope. AxioVision software was used for size measurements and image processing.

### Western blotting

Total protein (30 μg) was fractionated on 10% polyacrylamide gel and blotted onto a methanol-activated PVDF membrane. PVDF membrane was blocked overnight in blocking buffer (Supplementary Table 3) at 4°C. The blocking buffer was replaced with rabbit anti-FtsZ primary antibody (1:10000) solution for FtsZ or rabbit anti-RRF primary antibody (1:20000) solution for RRF. The membrane blots were washed and incubated with 1:10000 diluted anti-rabbit goat IgG (Sigma, Solna, Sweden) (Srivastava et al., 2016). The blots were washed and developed with X-ray film (Kodak) or chemiluminescence imager (ImageQuant LAS 4000) using Clarity Western ECL Substrate (Bio-Rad, Solna, Sweden).

### SYTO9/PI staining

SYTO9 and propidium iodide (PI) were prepared according to the manufacturer’s instructions [Live/Dead Bacterial Kit, Molecular Probes, Gothenburg, Sweden (Brantl, 2007)]. Cells in 50 μl of culture were stained with 0.1 mM SYTO9/PI mix for 15 min in dark at 25°C, washed with PBS solution, and images were taken with excitation and emission of 483/503 nm for SYTO9 and 485/630 nm for PI.

### Statistical analysis for significance

Statistical analysis of significance was performed between two data sets in a two-tailed *t*-test. The *p*-values range were indicated with asterisks (*p < 0.05, ^**^p < 0.01, ^***^p < 0.001).

## Results

### StfZ RNA is expressed at all growth phases

**FIGURE 1:**
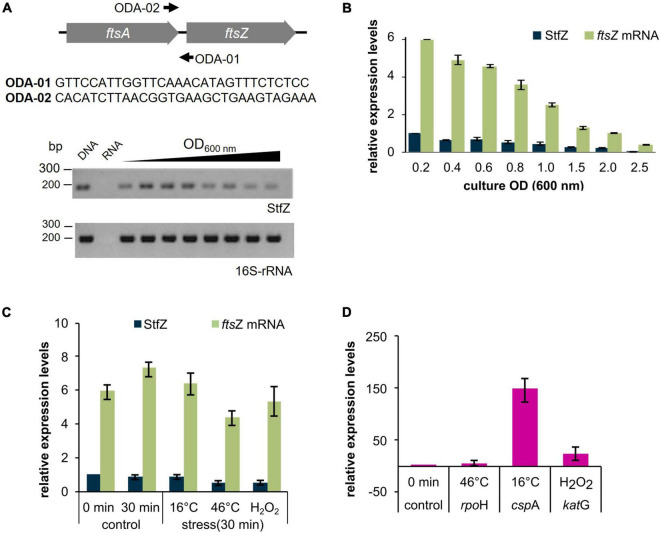
Detection and expression of StfZ RNA in stress conditions. RT-PCR for the detection of StfZ transcript. **(A)** The upper panel shows the location of the primers ODA-01 and ODA-02 used for StfZ RT-PCR and the sequence of the primers. Oligo ODA-02 was used for StfZ cDNA synthesis. The lower panel shows the RT-PCR products of StfZ and 16S rRNA after 30 cycles of amplification. G-DNA, genomic DNA as the positive control; RNA, RNA sample from 0.6 OD as the negative control; and cDNA from 0.2 to 2.5 OD cultures. The RT-PCR products were of 177 bp (StfZ) and 218 bp (16S-rRNA). **(B)** Bar-graph for the relative levels of the *ftsZ* and StfZ transcripts. Real-time PCR of *ftsZ* mRNA (green) and StfZ transcript (dark blue) from *E. coli* K12 cells from 0.2 to 2.5 OD cultures were performed on cDNA synthesised with ODA-06 oligo (for *ftsZ*) and ODA-02 oligo (for StfZ). Relative levels of *ftsZ* mRNA and StfZ are normalised to StfZ level from 0.2 OD. **(C)** Real-time PCR of *fts*Z mRNA (green) and StfZ (dark blue) from K12 cells. Cells were stressed for 30 min at 16°C, 46°C, and 5 mM of H_2_O_2_ exposure. The bar graph represents the levels of the transcripts to StfZ at 0 min. **(D)** Real-time PCR of *rpo*H, *csp*A, and *kat*G mRNA levels (markers for stress response) after 30 min of heat shock (Grossman et al., 1984), cold shock (Etchegaray et al., 1996), and oxidative stress (Erickson et al., 1987) response, respectively. Expression level from the uninduced culture was used as the expression control.

We determined the presence of StfZ transcript in *E. coli* K12 cells from 0.2 (early log phase) to 2.5 (late stationary phase) OD_600 *nm*_ (hereinafter called OD) cultures. RT-PCR analysis, performed using ODA-01 and ODA-02 primers located across the intergenic region of *ftsA* and *ftsZ*, showed the presence of 177 bp RT-PCR product from all the growth phases (Figure 1A). Cloning and sequencing of the StfZ RT-PCR product confirmed that StfZ is transcribed at all growth phases in a pattern of intensity that is growth phase dependent.

### Stoichiometric expression of StfZ RNA to *ftsZ* mRNA

To find the stoichiometry between the antisense StfZ RNA and its target sense *ftsZ* mRNA at different growth phases, their levels were determined using quantitative real-time PCR. The StfZ RNA level was highest at the early log phase (0.2 OD) and was maintained at a relatively high level till the mid-log phase (0.6 OD) (Figure 1B). Subsequently, its levels progressively decreased as the culture approached stationary phase (1.5 OD), where the level was about 5-fold lower compared to those in the 0.2 OD cultures. The StfZ level was further reduced at 2.5 OD (Figure 1B). The decrease in the StfZ RNA level was thus found to be growth phase dependent. This decrease in StfZ RNA correlated with the steady decrease in the level of its target, *ftsZ* mRNA (Figure 1B). Consistent with this correlation, the ratio between StfZ RNA to *ftsZ* mRNA was found to be always ∼1:6, irrespective of the growth phase. This indicated a coordinated expression of the sense and the antisense RNAs in a growth-phase-dependent manner.

### StfZ expression in stress conditions

Bacteria respond to stress conditions by expressing genes that provide a defensive mechanism to counter and/or survive under the stress. During heat shock, a set of proteins called, heat-shock proteins (HSPs) are expressed (Grossman et al., 1984), in cold, cold shock proteins (CSPs) are expressed (Etchegaray et al., 1996) and in oxidative stress, catalases/hydroperoxidases are expressed (Schellhorn, 1995). In *E. coli*, small RNAs like OxyS are expressed to protect against oxidative damage. It induces cell cycle arrest to allow DNA damage repair (Altuvia et al., 1997). We wanted to find out a stress condition that can influence the expression of StfZ and eventually cell division. We analysed the expression pattern of StfZ and *fts*Z mRNA after 30 min of heat-shock, cold-shock, and oxidative stress. Levels of StfZ did not show any significant difference under any of the conditions tested as compared to the unstressed samples (Figures 1C,D). There was about a 40% decrease in StfZ after 30 min of heat shock and H_2_O_2_ treatment. However, both the changes were not statistically different from the 0 min sample. Therefore, we conclude that heat shock and oxidative stress have only a minor effect on StfZ levels.

### Multiple transcripts of StfZ

**FIGURE 2:**
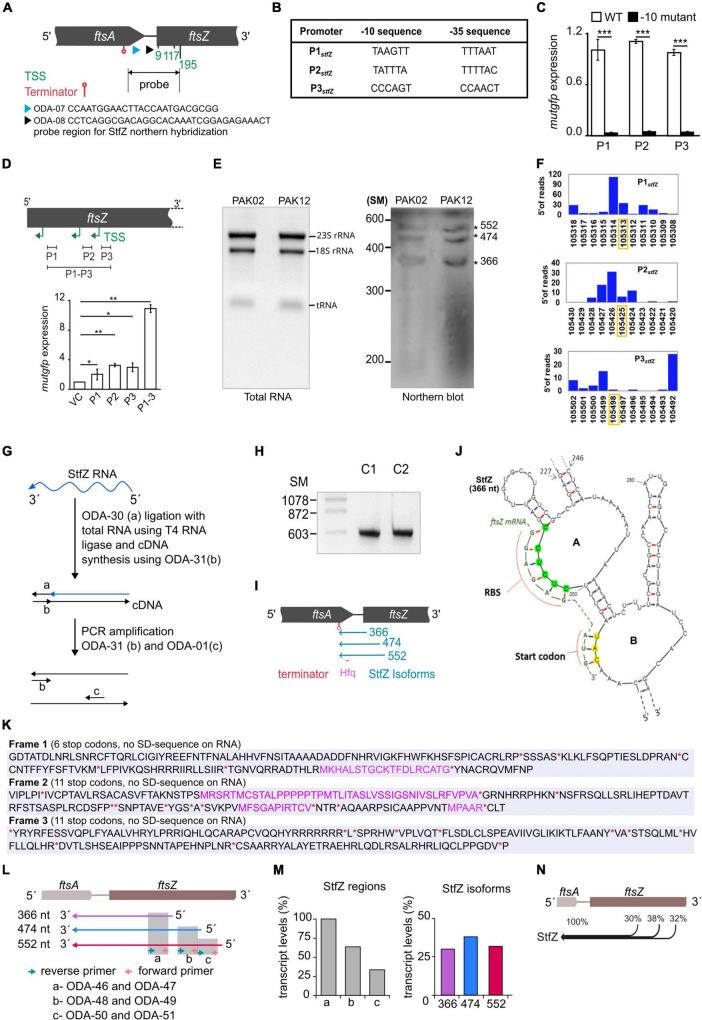
Identification of the 5′ end, the promoters, and the 3′ end of StfZ. **(A)** Schematic representation of the positions of the oligonucleotides used for primer extension analysis (PEA), which identified TSS sites and terminator. ODA–07 (blue), ODA–08 (black), TSS (green), and terminator (red). The double arrow line indicates the location of the probe used for northern blotting in 2E. **(B)** Putative promoters of the *stfZ* with their respective –10 and –35 sequences predicted from the PEA products. **(C)** Promoter assay using *mutgfp* as a reporter in pFPV27 vector. P1, P2, and P3 predicted promoters with native sequence (WT) (white bars) and respective –10 deletion mutant –10 mutant (black bars) were cloned upstream to *mutgfp*. Strain PAK05 (P1 wt), PAK07 (P2 wt), PAK09 (P3 wt), PAK06 (P1Δ-10), PAK08 (P2Δ-10), and PAK10 (P3Δ-10) were used. Y-axis indicates the relative expression. **(D)** Relative activity of *stfZ* promoters, P1, P2, and P3, individually and cumulative were analysed in PAK05, PAK07, PAK09, and PAK11 (P1 + P2 + P3) strains, respectively. The illustration (top) shows the cloned promoter regions (black horizontal lines). pFPV27 was used as the vector control. Bar graphs show the relative expression of *mutgfp* (y-axis) from different promoters (x-axis). The *p*-values range were indicated with asterisks (***p < 0.001). **(E)** Northern blot for PAK02 and PAK12 RNA samples. PAK02 and PAK12 were probed with a single-stranded RNA probe. RNA ladder was used as a size marker. The asterisks indicate three bands of expected sizes, 366, 474, and 552 nt, approximately. **(F)** Transcription start site (TSS) frequency bar-plot from the existing RNA seq data represents the surrounding region of the StfZ 5′ end identified by primer extension. *stfZ*-P1, *stfZ*-P2, and *stfZ*-P3 in this graph represent the promoters of TSS–9, TSS–117, and TSS–195. The Y-axis on each graph represents the number of reads starting on each position and the X-axis represents the genomic positions. RNA-Seq data was extracted from NCBI-SRA (accession number- SRX3413960). The orange box shows the TSS determined from PEA in this study. **(G)** Strategy for 3′ RACE to identify the 3′ end of StfZ RNA. **(H)** The approximate size of the 3′ RACE product is 600 bp. C1 and C2 are two biological replicates of 3′ RACE. SM, size marker in nucleotides. **(I)** Concise diagram indicating the location of StfZ isoforms at *fts*A-*fts*Z locus. Identified terminator position from 3′ RACE (red) predicted Hfq binding site (magenta), and different isoforms with their respective sizes (blue). **(J)** 5′ region of the predicted secondary structure of StfZ RNA (366 nt) from the Mfold web server. The *ftsZ* (yellow) ribosomal binding site (RBS) and of the AUG start codon complementary to the StfZ structure are shown next to the open loops, A and B, respectively. **(K)** *In silico* analysis of StfZ sequence for peptide reading frame. In black, peptides start without methionine; in violet, peptides start with methionine; and in red asterisk ‘*’ and ‘**’, stop codons. Ribosome binding sites are not present in the entire StfZ RNA sequence. **(L)** Primer map for real-time PCR to estimate the levels of individual StfZ isoforms. Isoforms 366, 474, and 552 are indicated as arrows below the gene locus of *ftsA-ftsZ* in violet, blue, and red, respectively. Green and orange arrows indicate the reverse and forward primers. Grey boxes, “a,” “b,” and “c,” show the coverage from the primers as mentioned in the lower panel. **(M)** Bar graphs for the transcript levels of StfZ regions and isoforms. The bar graph in the left panel shows the transcript levels detected from regions “a,” “b,” and “c” (grey bars). The relative amount was calculated with respect to the region “a” taken as 100%. The bar graph in the right panel shows the relative levels of three StfZ isoforms as percent transcript. Violet- StfZ 366, blue- StfZ 474, and red- StfZ 552. Y-axis, percent transcript levels. **(N)** Map of StfZ isoforms and their contribution to StfZ RNA pool. Black merging horizontal lines with reference to *ftsA*-*ftsZ* genomic locus (top) show isoforms with relative amounts of their transcripts.

For determining the 5′ and 3′ ends and thereby the length of StfZ RNA, primer extension assay (PEA), 3′ rapid amplification of cDNA ends (3′ RACE), and northern hybridisation were performed. PEA was performed using ODA-07 and ODA–08 primers (Supplementary Table 4) located in the intergenic region of *ftsA* and *ftsZ* (Figure 2A; Supplementary Figure 1A). We obtained three products from the extension of the ODA–07 primer. The first product was located at 9th, second at 117th, and third at 195th positions 3′ to *ftsZ* ATG start codon (Supplementary Figure 1B). Primer ODA–08 positioned downstream to ODA–07 binding site, gave two products, the one at the 117th and the other at the 195th positions 3′ to *ftsZ* ATG start codon (Supplementary Figure 1C). These products were overlapping with the products obtained from ODA–07, thereby confirming the authenticity of the PEA products. These three PE products were named TSS–9, TSS–117, and TSS–195, according to the distance from *ftsZ* 5′ end (Figure 2A). Subsequently, we compared the consensus sequences of −10 and −35 regions of *E. coli* promoters (Hawley and Mcclure, 1983; Lisser and Margalit, 1993; Mitchell et al., 2003) with the sequence in the region upstream of the respective 5′ end nt of the three respective PE products. Thus, the predicted promoter sequences for TSS–9, TSS–117, and TSS–195 were named P1, P2, and P3, respectively (Figure 2B). P1 and P2 showed −10 consensus with TATAAT and −35 consensus with TTGACA of the experimentally identified promoters of *E. coli* (Hawley and Mcclure, 1983; Lisser and Margalit, 1993; Hershberg et al., 2001; Mitchell et al., 2003). The predicted −10 and −35 sequences for the putative P3 promoter showed divergence (Figure 2B).

### The predicted promoters of StfZ RNA drive reporter gene expression

The −10 regions of bacterial promoters are crucial for the initial stages of sigma factor interaction. Transcription initiation drastically fails in the absence of the −10 element (Ruff et al., 2015; Browning and Busby, 2016). This characteristic feature has been used to validate and map bacterial promoters. Taking the same approach to validate the predicted promoters, we constructed Δ-10 promoter constructs (pDA3, pDA5, and pDA7) (Supplementary Table 2), with *mutgfp* as the reporter gene, and compared its expression from the respective native promoter constructs (pDA2, pDA4, and pDA6) (Supplementary Table 2).

Deletion of predicted −10 elements of the three putative promoters showed about a 20–fold reduced expression of *mutgfp* (Figure 2C). The transcriptional activity of the three predicted promoters and its abrogation in the −10 deletion mutants validated the authenticity of the promoters. Individual activity of the putative promoters showed different significant levels of expression. Whereas a significant level of cumulative expression was observed from the combined P1-P2-P3 promoter construct, pDA8 (Supplementary Table 2; Figure 2D). These observations implied transcription of *stfZ* from three independent promoters producing three isoforms of StfZ. It was of interest to note that the P1 promoter region corresponded to the previously predicted promoter for StfZ (Dewar and Donachie, 1993).

To specifically study the activity of the promoters, we created mutant versions of the −10 elements of the three promoters and compared their activity. The selected mutations were substitution mutations in the FtsZ protein-coding sequence (Supplementary Figure 2A). Promoter activity was analysed using *mutgfp* reporter assay. We found that the sequences of the −10 mutant promoters did not show any significant difference in *mutgfp* expression as compared to the respective WT promoter sequence (Supplementary Figure 2B). This result shows that the promoter sequences are capable of initiating transcription even when minor changes are introduced in their −10 elements, as reported (Raghavan et al., 2012).

### Northern blotting shows three isoforms

Northern blotting against StfZ RNA, which was performed using total RNA from *E. coli* K12 (wt) and PAK12 transformant carrying the cloned *stfZ* gene spanning all the three isoforms (Supplementary Table 1), showed bands in the range of ∼350, ∼450, and ∼500 nts (Figure 2E). The consistent presence of the expected three bands despite high stringency washes with 0.1x SCC and 0.1% SDS at 55°C indicated their authenticity. The low intensity of the bands suggested a low level of StfZ expression, which could be observed by RT-PCR (see Figure 1A).

Based on our experimental analysis, we examined the existing RNA-Seq data to know whether three StfZ transcripts were detected before this study. RNA-Seq data were extracted from the NCBI SRA database (accession number–SRX3413960) (Livingstone et al., 2018) and analysed for the genomic positions of TSSs (Transcription Start Sites) in the *stfZ* region. TSSs were plotted as bar graphs along the DNA sequence. The TSSs of StfZ were detected wherefrom TSS-9, TSS-117, and TSS-195 RNAs were transcribed (Figure 2F). However, the genomic positions did not match precisely with the RNA-Seq + 1 TSS from PEA. Thus, the slight variation in the RNA-Seq TSS sites suggested only a close possibility of TSS but not the exact position of TSS. Therefore, we relied on PEA data more than the RNA-Seq data.

Additionally, to verify the exact ends of the isomers and to find out whether it is a processed RNA product, we performed circular RACE with and without Tobacco Acid Pyrophosphatase (TAP) treatment of RNA (McGrath, 2011). TAP removes 5′ cap of RNA therefore processed RNA can be detected in circular RACE without TAP treatment but not a capped RNA. Using this method, we did not find any amplification from the StfZ region under any condition, which indicated that StfZ RNA was not a processed product of any primary RNA.

### StfZ RNA 3′ end extends to upstream of *ftsZ*

Antisense RNA function depends on their coverage and location on the target RNA. Therefore, after determining the 5′ ends of StfZ transcripts, we performed 3′ RACE to find out the 3′ end(s) of the transcripts. For this, the ribominus RNA fraction (devoid of ribosomal RNAs) was ligated to an adaptor oligo, ODA-30, and the cDNAs were synthesised using complementary oligo ODA–31, as indicated in the cartoon (Figure 2G; Supplementary Table 4). The cDNA product was amplified with ODA–31 and ODA–01 primers to get the PCR product of ∼600 bp (Figure 2H). Biological replicates C1 and C2 of PCR amplified products were cloned in plasmid pDA1 and sequenced (Figure 2H; Supplementary Table 2). Sequencing the replicates from both ends showed 357th nt 5′ upstream of *ftsZ* as the common 3′ ends of all the three isoforms. The entire sequence of the three isoforms encompassed the ribosome binding sequence (RBS) of *ftsZ*, the entire *ftsA-ftsZ* intergenic region, and 297 nts on the 3′ end of *ftsA* gene located 5′ upstream of *ftsZ* (Figure 2I). From the three different transcription initiation sites, StfZ isoforms are produced as 366, 474, and 552 nt long RNAs (Figure 2I). These sizes corresponded to the sizes of the three PEA products and of the three bands in the northern blot (Supplementary Figure 1; Figure 2E). The sequences were deposited in Bankit database with accession numbers; stfZ_366 KX852304, stfZ_474 KX852303, and stfZ_552 KX852302.

### Features of StfZ RNA

Interaction of antisense RNA with its target RNA involves the formation of a “kissing complex” which eventually makes a stable RNA–RNA complex (Gerhart et al., 1994; Lease and Woodson, 2004; Brantl, 2007). Therefore, we predicted a secondary structure of the 366-nt long StfZ isoform using Mfold (Zuker, 2003). The structure showed the presence of two successive loops A and B with 5′ CUCUCC 3′ (complementary region of *ftsZ* mRNA RBS, 5′ GGAGAG 3′) and 5′ CAU 3′ (complementary to *ftsZ* initiation codon 5′ AUG 3′) (Figure 2J). The StfZ sequence also has 5′ AATAATA 3′ sequence, which resembled the potential consensus sequence for the binding of Hfq (5′ AAYAAYAA 3′) (Lorenz et al., 2010). It is located at 156 nt upstream from the 3′ end of the StfZ transcript and it shares a complementary region of *ftsA* (Figure 2I). StfZ RNA sequence has multiple stop codons (6 in reading frames 1, and 11 each in reading frames 2 and 3) and no ribosomal binding sites (RBS). There are small peptide open reading frames but no RBS for translation (Figure 2K).

### StfZ isoforms are transcribed in comparable levels

After estimating the size of all the isoforms, we investigated the contribution of expression from individual isoforms. To do so, we used three sets of primers in such a way that it covers different regions of StfZ isoforms (Figure 2L, top panel). Real-time PCR was performed on these regions using the cDNAs prepared from the reverse primers, ODA-47, ODA-49, and ODA–51, for the 366 nt, 474 nt, and 552 nt isoforms, respectively. The level of the transcripts was calculated from three regions. Region “a” covers all three isoforms thus it was used as the 100% level. Region “b” covers two isoforms, 474 and 552. Region “c” covers only isoform 552. The relative level of region “a” was the highest, and region “c” was the lowest (Figure 2M, left panel). Using the maps from Figure 2K, we calculated the contributions of each isoform and found that the relative level of isoform 366 is 30%, isoform 474 is 38%, and isoform 552 is 32% (Figure 2M, right panel). Therefore, different isoforms contribute equally to StfZ RNA pool (Figure 2N).

### StfZ RNA target is *ftsZ* mRNA

**FIGURE 3:**
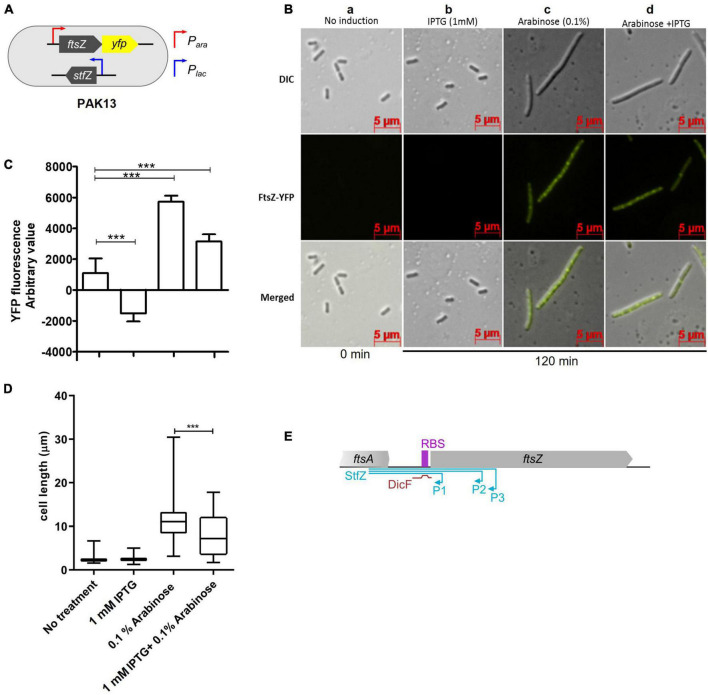
Inhibition of *ftsZ-yfp* mRNA translation by StfZ. PAK13 cells were induced with either 1 mM IPTG or/and 0.1% arabinose at 37°C for 120 min. **(A)** Schematic presentation of PAK13 strain with *P_*ara*_-ftsZ-yfp* and *P_*lac*_-stfZ* constructs. **(B)** Cells were imaged before induction (0 min) and after induction for 120 min. Columns: (a) negative control without induction of the culture; (b) positive control for StfZ RNA expression, induced with IPTG; (c) positive control for FtsZ-YFP expression, induced with arabinose; (d) experimental sample, induced for the co-expression of StfZ RNA and *ftsZ*-*yfp* fusion mRNA. Top row: DIC images; middle row: FtsZ-YFP images; and lower row: merged images. **(C)** Quantitation of the YFP fluorescence as arbitrary values for 0 min and 120 min post-induction from 200 μl culture. **(D)** Box chart for cell size at 0 min and 120 min of induction (n > 300). **(E)** Diagram representing the span of StfZ and DicF antisense RNA on *ftsZ* sequence. The *stfZ* (blue line) covers the complete intergenic region and a significant portion of ftsZ while DicF (brown line) partially covers the intergenic region. Statistical significance is indicated with asterisks (***p < 0.001).

The sequence features of StfZ RNA and its location on the strand complementary to *ftsZ* mRNA reading frame revealed that it can function as an antisense RNA specific to *ftsZ* mRNA. Therefore, the effect of StfZ RNA overexpression on *ftsZ*-*yfp* translation was tested to verify its target specificity and to document the physiological changes brought about by StfZ overexpression. This method allowed measurement of the effect of the antisense RNA against its target *ftsZ* mRNA by fluorescence microscopy or directly by YFP fluorescence from bacterial cells. In principle, binding of StfZ RNA to *ftsZ-yfp* should rescue FtsZ-YFP overexpression phenotype (cell elongation/filamentation) and reduce YFP fluorescence in the PAK13 strain (Figure 3A). PAK13 contains, pDA9 (423 bp region of *stfZ*, spanning all the three isoforms, cloned under *P*_*lac*_) and pBAD33-*ftsZ*-*yfp* (a gift from W. Margolin, *ftsZ*-*yfp* cloned under *P*_*BAD*_ promoter). The culture was induced at 0.6 OD with either 0.1% arabinose (for *ftsZ-yfp* mRNA) or 1 mM IPTG (for StfZ RNA) or with both the inducers simultaneously. Expression of FtsZ-YFP was measured at the fluorescence level in the culture and single-cell level by microscopy. Multiple FtsZ-YFP rings and a high level of YFP fluorescence were observed in the arabinose-induced cells due to overexpression of FtsZ-YFP (Figure 3B, c, d and Figure 3C, third bar). A high level of FtsZ-YFP interfered with the division process and induced cell elongation/filamentation (Figure 3D). The co-induction of StfZ RNA along with *ftsZ* mRNA rescued the cells from elongation/filamentation with a significant reduction in the cell length (Figure 3D). Inhibition of *ftsZ-yfp* translation could be inferred from the reduction in the YFP fluorescence level in the FtsZ-YFP induced cells (Figure 3C, second bar). This effect was also visible in the cells that were induced only with IPTG, which showed induction of low levels of FtsZ-YFP (Figure 3C, first bar). These cells did not filament as the overexpressed StfZ might have been engaged in the interaction mostly with *fts*Z-*yfp* mRNA and probably to a low extent with the native *fts*Z mRNA. Thus, the sequestration of StfZ by the *ftsZ-yfp* mRNA might have effectively prevented its interaction with *ftsZ* mRNA and the consequential division inhibition and elongation/filamentation. The reduction in the YFP fluorescence in these cells supported this possibility (Figure 3C, second bar). These results suggested that StfZ interacts with *ftsZ* mRNA. There is a functional overlap between DicF and StfZ RNAs as they share the target region near the *ftsZ* RBS sequence (Figure 3E). However, *E. coli* K12 and JM109 strains did not contain DicF RNA as found using RT-PCR (data not shown), ruling out any interference by DicF RNA in these experiments.

### StfZ RNA influences FtsZ level and thereby cell division

**FIGURE 4:**
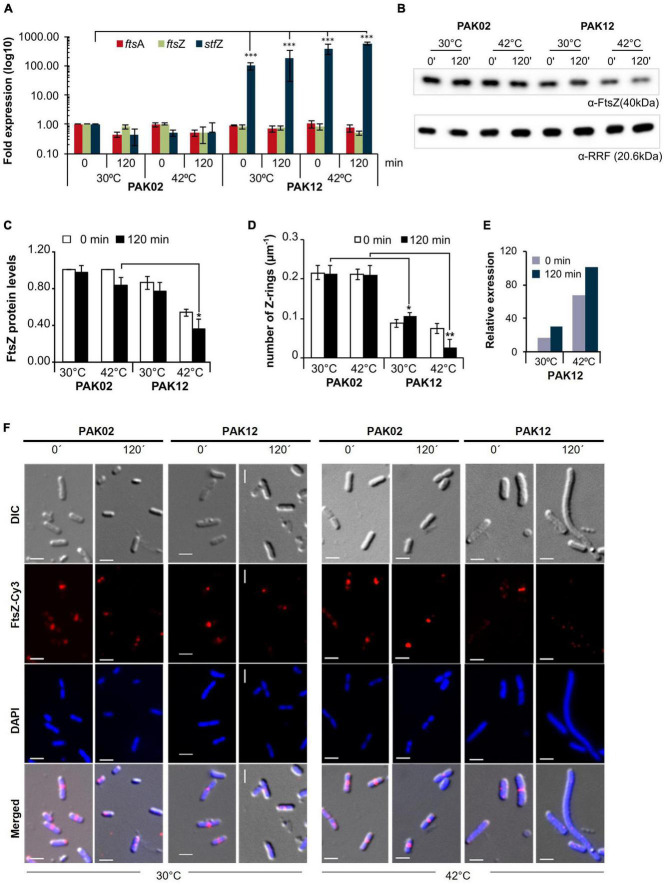
StfZ over induction decreases FtsZ level and blocks cell division. **(A)** Real-time PCR for StfZ, *fts*Z, and *fts*A mRNA at 30 and 42°C in PAK02 (vector control) and PAK12 (StfZ expression) strains. **(B)** Western blot analysis for FtsZ level and equal loading profile of RRF protein (Ribosome-recycling factor). **(C)** Bar-graph for the quantitation of FtsZ from western blot. FtsZ level was calculated to PAK02 sample from 0 min at 30°C. **(D)** Bar-graph showing ratio of FtsZ rings per micron of cell length (n > 100). Cells were fixed after 0 min and 120 min of induction and immunostained for FtsZ. The total number of Z-ring was divided by the cumulative cell length of the population. **(E)** Estimation of relative RNA expression of StfZ compared to *ftsZ* mRNA at 30 and 42°C. Bar-graph showing RNA levels of StfZ RNA from real-time PCR data [data from panel **(A)**] normalised with *ftsZ* level by 6-fold. **(F)** Immunostaining for FtsZ in PAK02 and PAK12 cells from 30 and 42°C cultures. For each temperature (bottom), left panel PAK02 (vector control) and right panel PAK12 (StfZ expression) at 0 min and 120 min are shown. FtsZ-Cy3 (red) and DAPI (blue). Scale bar, 5 μm. Statistical significance is indicated with asterisks (*p < 0.05, **p < 0.01, ***p < 0.001).

Unlike a large number of *trans*-antisense RNAs that are encoded at loci far away from the loci coding for their target RNAs (Majdalani et al., 2004; Papenfort et al., 2009; Guo et al., 2014), the sequence of StfZ RNA is complementary to the reading frame of its target RNA, *ftsZ*, which is essential for cell division. Therefore, the generation of *stfZ* knockout mutant and/or its promoter mutations, which would fall on *ftsZ* reading frame, could not be taken up to determine the physiological effect of the lack of expression of StfZ from the native locus. Therefore, we overexpressed StfZ to study its influence on FtsZ protein level, cell division, and growth. A 423 bp region of *stfZ*, which spans across the promoters and reading frames of all the three isoforms, was cloned and expressed from PAK12 (P*_*lac–*_stfZ*) strain. The strains PAK02 (vector control) and PAK12 (Supplementary Table 1) were induced with 1 mM IPTG for 2 h at 30 and 42°C. A higher induction temperature of 42°C was used as per DicF *trans*-antisense RNA experiment, where the translation of FtsZ was blocked only at 42°C but not at lower temperatures probably due to its strong secondary structures (Tétart and Bouché, 1992). Upon induction of *stfZ* in the PAK12 cells, the levels of StfZ RNA increased 180-fold and 600-fold, at 30 and 42°C at 120 min, respectively, as compared to the levels of the endogenous StfZ RNA in the PAK02 cells (Figure 4A). Quantitation of the levels of *fts*Z and *fts*A mRNAs from the PAK02 and PAK12 cells did not show any significant difference upon *stfZ* induction at either temperature even for 120 min indicating that *stfZ* induction did not degrade *ftsZ* mRNA (Figure 4A). However, at 120 min post-induction of *stfZ* at 42°C, the levels of FtsZ protein were only 17% in the PAK12 cells (Figure 4B, upper panel and Figure 4C). The change in the FtsZ levels at 30°C was not significant (Figure 4C). The loading control is shown for RRF (Figure 4B, lower panel). Thus, the target sense RNA did not get degraded in the case of StfZ RNA-*ftsZ* mRNA interaction, unlike in many cases (Dühring et al., 2006; Giangrossi et al., 2010; Lee and Groisman, 2010; Bordoy and Chatterjee, 2015).

The influence of reduced FtsZ level on cell division was examined by determining the ratio of FtsZ-rings per micrometre length of the cells by counting the number of cells with immuno-stained FtsZ rings. A higher number of FtsZ-rings per micron would show a higher rate of division while a lesser number would show a reduced frequency of division. In PAK02, the cell division ratio was comparable at 30 and 42°C. On the contrary, in the PAK12 cells, the ratio was significantly reduced at 30°C and even more so at 42°C, indicating a reduction in the number of cells undergoing division (Figure 4D). To calculate the amount of StfZ RNA needed to influence cell division significantly, we extrapolated the data from Figure 4A. We calculated the relative levels of StfZ RNA under overexpressed conditions with respect to native *ftsZ* mRNA levels. We found that 30- to 100-fold higher StfZ RNA expression at 30 and 42°C, respectively, as compared to *ftsZ* mRNA, caused cell division defects (Figure 4E). This data is reflected in the images of FtsZ-immunostained PAK02 and PAK12 cells at 30 and 42°C at 0 min and 120 min (Figure 4F). At 30°C, higher expression of StfZ did not cause any filamentation while resulting in cell elongation after 120 min at 42°C in PAK12 cells. Cells with vector control did not show any effect at both temperatures (Figure 4F).

At 37°C also, induced expression of StfZ for 120 min showed about a 1.75-fold reduction in the FtsZ level as compared to the 0 min sample (Supplementary Figure 3A). Commensurate with this reduction, the proportion of the cells with FtsZ-ring also decreased significantly from ∼60 to ∼40% in 120 min (Supplementary Figure 3B).

Measurement of optical density of the cells carrying uninduced and induced StfZ RNA at 42°C showed a significant increase in PAK02 and PAK12 mass (Supplementary Figure 3C). However, PAK12 mass was significantly low at 120 min compared to PAK02 indicating inhibition of cell division and the consequential lack of increase in the cell number. The higher OD of PAK02 at 120 min might be from the cell number increase due to cell division. Colony-forming unit (CFU) at 120 min showed a significant increase in the PAK02 population while the PAK12 cells did not show a significant increase between 0 and 120 min (Supplementary Figure 3D). The CFU data corroborated with the cell mass data that the lack of increase in cell mass in PAK12 was a result of cell division inhibition and that the increase in cell mass in PAK02 was due to an increase in the cell number by cell division. SYTO9/PI staining confirmed that 120 min of StfZ induction did not affect the cell viability (Supplementary Figure 3E).

To understand the effect of the interaction between StfZ RNA and *ftsZ* mRNA, we designed an StfZ variant without its *ftsZ*-RBS complementary sequence (StfZ-ΔRBSc; PAK14; Supplementary Table 1). It was overexpressed by induction identical to StfZ overexpression at 30 and 42°C. Like the overexpressed StfZ RNA, the overexpressed StfZ-ΔRBSc RNA did not affect *ftsZ* mRNA stability as it did not decrease the levels of the target RNA (Supplementary Figure 4A), which meant that it did not degrade *ftsZ* mRNA. Further, like the StfZ RNA, StfZ-ΔRBSc RNA also affected the levels of FtsZ protein (Supplementary Figures 4B,C). The reduction in the levels of FtsZ reduced the number of cells undergoing cell division (i.e., the number of cells with FtsZ-rings) after 120 min of induction at 42°C thereby affecting cell division (Supplementary Figures 4D,E). However, the number of Z-rings per micrometre of PAK14 cells did not show a statistically significant difference compared to that of the PAK12 cells (Supplementary Figure 4E). Thus, the effect of StfZ-ΔRBSc RNA on FtsZ and cell division was relatively similar to StfZ.

Thus, taken together, the *cis*-encoded StfZ RNA emerges as a novel factor involved in the maintenance of *ftsZ* mRNA levels available for translation, and hence of FtsZ protein levels, at all phases of growth and cell division in *E. coli*.

## Discussion

### Features of structure and expression of StfZ RNA

This study showed for the first time the complete sequence identity of StfZ RNA, with its 5′ and 3′ ends, three promoters transcribing them, stoichiometric expression with respect to *ftsZ* mRNA, and its ability to reduce FtsZ levels when overexpressed (Figures 2, 4). Overexpressed levels of StfZ imposed cell division block, resulting in cell elongation and filamentation. The predicted StfZ structure showed its possible initial interaction with *ftsZ* mRNA at the RBS site to form a “kissing complex” followed by a complete stable duplex (Figure 2J; Gerhart et al., 1994; Lease and Woodson, 2004; Brantl, 2007). However, the deletion of the RBS interacting region from StfZ showed a possibility of another open loop interaction that also reduced FtsZ levels (Supplementary Figure 4). Also, FtsZ protein reduction was not due to a decrease in the *ftsZ* mRNA levels unlike in the case of many sense-antisense RNA interactions (Figure 4A; Dühring et al., 2006; Giangrossi et al., 2010; Lee and Groisman, 2010; Bordoy and Chatterjee, 2015). StfZ RNA lacks the ribosome binding site (RBS) consensus sequence to translate any possible small ORF. From the existing proteomics data, we did not find any evidence of peptide that matches the StfZ region. This ruled out the possibility of StfZ coding for any short peptide, unlike the possibility predicted in the earlier study (Dewar and Donachie, 1993). The expression of StfZ throughout the entire growth phase showed growth-phase-dependent expression. Its levels at 1/6th of the proportion of *ftsZ* mRNA indicated that it may be involved in the fine regulation of *ftsZ* mRNA levels available for translation during different stages of growth, as per the growth-phase-specific demand in the bacteria. The higher levels of both *ftsZ* mRNA and StfZ RNA during early phases of active growth and their proportionate decrease during phases of reduced growth are indicative of the “synthesis-as-per-demand” mode of expression of FtsZ. However, the StfZ levels did not change during the 30 min of heat shock, cold shock, or oxidative stress (Figures 1C,D). This does not rule out the possibility of some other stress conditions under which a significant change may occur in the StfZ expression or the 1:6 ratio of StfZ to *ftsZ* mRNA. From the real-time PCR data, we extrapolated the stoichiometry between StfZ and *ftsZ* mRNA (Figure 4E) and found that StfZ could influence FtsZ levels at 30-fold higher expression compared to *ftsZ* mRNA, at 30°C in 120 min. A further increase to 100-fold (at 42°C, 120 min) caused more severe division inhibition. Such levels of StfZ RNA may be unnatural and we did not find such levels of StfZ at any phase of growth. Nevertheless, it indicated that the effects of StfZ RNA on *ftsZ* mRNA levels, and hence on FtsZ protein levels, are concentration dependent, which may mean a smaller increase affects cell size while a higher amount inhibits cell division. Therefore, it was imperative on the part of the cell to maintain the StfZ:*ftsZ* ratio at 1:6 at all phases of growth and cell division, which was what we observed.

The effect of StfZ RNA on *ftsZ* mRNA was higher at higher temperature (42°C) as in the case of DicF RNA (Bouché and Bouché, 1989). We speculated that higher temperature might have helped to melt the secondary structure which was essential for its interaction with the target RNA to form the “kissing complex.”

### Isoforms of StfZ RNA

Many *trans*-antisense RNAs have isoforms that are most often the processed forms of a primary transcript expressed from a single promoter. Some of such *trans*-antisense RNAs are the DicF (Bouché and Bouché, 1989), ArcZ (Papenfort et al., 2009; Soper et al., 2010), RprA (Majdalani et al., 2004), and MicL (Guo et al., 2014) RNAs of *E. coli.* On the contrary, in the case of StfZ RNA, the loss of activity of the −10 deletion mutants of the three promoters, predicted based on the 5′ end identification using PEA, validated the authenticity of the predicted promoters and the existence of three isoforms (Figure 2C). The transcriptional activity of the three predicted promoters and its abrogation in the respective Δ-10 constructs indicated that the predicted promoter sequences were capable of initiating transcription and therefore the primer extension products were not a result of a processed product. The −35 sequence (CCAACTT) of P3 promoter was interesting as it showed consensus with the −35 sequence (GGAACTT) of *rpoEp*3 gene of *Salmonella enterica serovar Typhimurium* (Skovierova et al., 2006; see Figure 2B). Many antisense RNA promoters of enteric bacteria do not show conservation in the −10 and −35 sequences (Raghavan et al., 2012). However, it was interesting to see the StfZ promoters also fall into the same category. We found that the P3 promoter would be one such promoter. It was also interesting to see all three promoters in the cloned format having similar strength in the plasmid context (Figure 2D). Additionally, real-time PCR from different regions of StfZ showed that the individual isoforms are expressed at similar levels (Figures 2M,N). This similarity did not correlate with the differences in the intensities of the three PEA products (see Supplementary Figure 1). This incongruity between the PEA data and the promoter assay data is not surprising as bacterial promoters showing different activities at different locations, such as in the genomic context or as individual clones in a plasmid, have been reported in many instances (Cases and de Lorenzo, 2005; Davis et al., 2011; Hocine et al., 2015; Srivastava et al., 2016). Further, the presence of three potential primer extension products or three bands in the northern blot could not alone conclude the existence of three promoters for StfZ RNA. Further, the *cis*-encoded nature of StfZ RNA to the essential gene *ftsZ* also did not permit their conclusive verification by mutating the promoters one at a time in the genome and checking for the decrease in the levels of StfZ RNA and consequential increase in FtsZ levels as it would have adversely affected the expression of the sense RNA causing lethality. Thus, studies of *cis*-encoded antisense RNAs have been possible only through overexpression, such as in the case of *ureB cis*-encoded antisense RNA against *ureAB* mRNA (Wen et al., 2011). Another example of a *cis*-encoded antisense RNA that exists in three isoforms is the *cis*-encoded GadY RNA, which regulates acid response genes in *E. coli* (Opdyke et al., 2004). Like in the case of StfZ RNA, all three isoforms of GadY RNA were detected at all growth phases in a growth-phase-dependent manner (Opdyke et al., 2004).

### The probable role of StfZ

It may be stated that differential expression of StfZ RNA from multiple promoters may help fine-tune the levels of *ftsZ* mRNA available for translation. It helps by controlling fluctuations in the FtsZ protein levels which is essential for proper cell division (Ward and Lutkenhaus, 1985; Bi and Lutkenhaus, 1990). Fine-tuning by StfZ seems to be a logical necessity for the cells to maintain a critical level of FtsZ, expressed from multiple promoters and through various other mechanisms (Vicente et al., 1998; Dewar and Dorazi, 2000). Differential expression of StfZ may also be important under certain stress conditions, which would be other than those that we tested. However, it is also possible that only under normal growth conditions, StfZ may be involved in the fine tuning of *ftsZ* mRNA levels and may not be under any stress conditions. Nevertheless, from the level of its expression being commensurate with the level of *ftsZ* mRNA at 1:6 ratio, it is tempting to speculate that the level of StfZ RNA would change in concert with *ftsZ* mRNA levels to keep the translatable *ftsZ* mRNA available at the required level at every phase of growth and cell division. This brings up the exciting proposition of the co-ordinated regulated expression of StfZ RNA and *ftsZ* mRNA genes. Future research in this direction would reveal the mechanisms behind StfZ RNA-mediated maintenance of the levels of *ftsZ* mRNA available for translation and hence of the levels of the essential cytokinetic protein, FtsZ, at all phases of growth and cell division. This will also open up questions on what other sense and *cis*-antisense genes are regulated similarly.

## Data availability statement

The datasets presented in this study can be found in online repositories. The names of the repository/repositories and accession number(s) can be found in the article/Supplementary material.

## Author contributions

PA, DA, DS, RN, and KJ conceived and designed the experiments. DA, DS, RN, and KJ performed experiments. PA, DA, DS, RN, KJ, and NM analysed the data and contributed reagents, materials, and analysis tools. PA, DA, DS, and RN wrote the manuscript. All authors contributed to the article and approved the submitted version.
