# Supplementary material for: Complete identity and expression of StfZ, the cis-antisense RNA to the mRNA of the cell division gene ftsZ, in Escherichia coli
Source: Front Microbiol. 2022 Oct 19;13:920117. doi: 10.3389/fmicb.2022.920117 (PMC9628754; doi:10.3389/fmicb.2022.920117)
Supplement: Supplementary file 1 [file Data_Sheet_1.PDF]

Supplementary Table S1 List of bacterial strains used in the study

Strain	Genotype	Plasmids	References
E. coli K12, Wild type *(wt)*	(F^+ lambda+)		(Blattner et al., 1997)
E. coli JM109	*recA1, supE44 endA1 hsdR17 gyr96 relA1 thiΔ(lac-proAB) F'[traD36proAB*$^+$ *lacI*^{q} *lacZ ΔM15]*		(Yanisch-Perron et al., 1985)
PAK01	JM109, pBS(KS)	pBS(KS)	This study
PAK02	JM109, pBS(KS)	pBS(KS)	This study
PAK03	JM109, *stfZ* 3' RACE	pDA1	This study
PAK04	JM109, pFPV27 (mutgfp)	pFPV27	This study
PAK05	JM109; $P1_{stfZ}$-*mutgfp*	pDA2	This study
PAK06	JM109; $P1\Delta\text{-}10_{stfZ}$-*mutgfp*	pDA3	This study
PAK07	JM109; $P2_{stfZ}$-*mutgfp*	pDA4	This study
PAK08	JM109; $P2\Delta\text{-}10_{stfZ}$-*mutgfp*	pDA5	This study
PAK09	JM109; $P3_{stfZ}$-P3-*mutgfp*	pDA6	This study
PAK10	JM109; $P3\Delta\text{-}10_{stfZ}$-*mutgfp*	pDA7	This study
PAK11	JM109; $P1\rightarrow3_{stfZ}$-*mutgfp*	pDA8	This study
PAK12	JM109; pBSKS/P_{lac}-*stfZ*	pDA9	This study
PAK13	JM109, pBAD33-*ftsZ-yfp;* P_{lac}-*stfZ*	pDA9, pBAD33	This study
PAK14	JM109, pBSKS/P_{lac}-*stfZ*-ΔRBSc	pDA10	This study
PAK15	JM109; $P1mut_{stfZ}$-*mutgfp*	pDA11	This study
PAK16	JM109; $P2mut_{stfZ}$-*mutgfp*	pDA12	This study
PAK17	JM109; $P3mut_{stfZ}$-*mutgfp*	pDA13	This study
