# Supplementary material for: Complete identity and expression of StfZ, the cis-antisense RNA to the mRNA of the cell division gene ftsZ, in Escherichia coli
Source: Front Microbiol. 2022 Oct 19;13:920117. doi: 10.3389/fmicb.2022.920117 (PMC9628754; doi:10.3389/fmicb.2022.920117)
Supplement: Supplementary file 2 [file Data_Sheet_2.PDF]

Supplementary Table S2 List of plasmids used in the study

Plasmids	Description	References
pBS(KS)	Cloning & expression vector	Alting-Mees et al, 1989
pFPV27	Promoter probe vector, *mutgfp* for *E. coli,ColE ori,* kan^R	Valdivia et al, 1996
pDA1	pBS(KS); *stfZ* 3' RACE, amp^R	This study
pDA2	Derivative of pFPV27, $P1_{stfZ}$*-mutgfp, kanR*	This study
pDA3	Derivative of pFPV27, $P1\Delta\text{-}10_{stfZ}$*-mutgfp,* kan^R	This study
pDA4	Derivative of pFPV27, $P2_{stfZ\text{-}}$*mutgfp,* kan^R	This study
pDA5	Derivative of pFPV27, $P2\Delta\text{-}10_{stfZ}$*-mutgfp,* kan^R	This study
pDA6	Derivative of pFPV27, $P3_{stfZ}$-P3-*mutgfp,* kan^R	This study
pDA7	Derivative of pFPV27, $P3\Delta\text{-}10_{stfZ}$*-mutgfp,* kan^R	This study
pDA8	Derivative of pFPV27, $P1\rightarrow 3_{stfZ}$*-mutgfp,* kan^R	This study
pDA9	Derivative of pBS(KS), P_{lac}*-stfZ,* amp^R	This study
pDA10	Derivative of pBS(KS), P_{lac}.stfZ-ΔRBSc	This study
pBAD33-*ftsZyfp*	Derivative of pBAD33, P_{ara}-Ec-*ftsZyfp,* amp^R,kan^R	W. Margolin
pDA11	Derivative of pFPV27, $P1mut_{stfZ}$*-mutgfp,* kan^R	This study
pDA12	Derivative of pFPV27, $P2mut_{stfZ}$*-mutgfp,* kan^R	This study
pDA13	Derivative of pFPV27, $P3mut_{stfZ}$*-mutgfp,* kan^R	This study
