# Supplementary material for: Complete identity and expression of StfZ, the cis-antisense RNA to the mRNA of the cell division gene ftsZ, in Escherichia coli
Source: Front Microbiol. 2022 Oct 19;13:920117. doi: 10.3389/fmicb.2022.920117 (PMC9628754; doi:10.3389/fmicb.2022.920117)
Supplement: Supplementary file 3 [file Data_Sheet_3.PDF]

Supplementary Table S3 List of reagents used in the study.

Method	Buffer	Composition
RNA Preparation	Lysis buffer	100 mM Sodium acetate (pH 5.2), 10 mM EDTA (pH 8.0), 1% (w/v) SDS,100 mM NaCl, 100 mM β-mercaptoethanol, and 5 mM vanadyl ribonucleoside complex
c-DNA preparation	Reaction buffer	50 mM Tris-HCl (pH 8.3), 75 mM KCl, 3 mM $MgCl_2$, and 10 mM DTT
PNK reaction buffer	PNK 1x buffer	50 mM Tris-HCl (pH 7.6), 10 mM $MgCl_2$, 5 mM DTT, and 0.1 mM spermidine
Northern hybridisation	Pre-hybridisation buffer	7% SDS, 200 mM Na_2HPO_4 (pH 7.0) and 5 µg/ml salmon sperm DNA
	Hybridisation buffer	7% SDS, 200 mM Na_2HPO4 (pH 7.0) and Biotin labeled probe
Immunostaining	PBS	137 mM NaCl, 2.7 mM KCl, 10 mM Na_2HPO_4 (pH 7.4), 2 mM KH_2PO_4
	PBST solution	137 mM NaCl, 2.7 mM KCl, 10 mM Na_2HPO_4 (pH 7.4), 2 mM KH_2PO_4, Tween 20 (0.1%)
Western blot	PBST	137 mM NaCl, 2.7 mM KCl, 10 mM Na_2HPO_4 (pH 7.4), 2 mM KH_2PO_4, Tween 20 (0.1%)
	blocking buffer	5% w/v skimmed milk, 1x PBST
