# Supplementary material for: Complete identity and expression of StfZ, the cis-antisense RNA to the mRNA of the cell division gene ftsZ, in Escherichia coli
Source: Front Microbiol. 2022 Oct 19;13:920117. doi: 10.3389/fmicb.2022.920117 (PMC9628754; doi:10.3389/fmicb.2022.920117)
Supplement: Supplementary file 4 [file Data_Sheet_4.PDF]

Supplementary Table S4- List of oligonucleotides used in the study

Oligo number	Oligo name	Sequence (5' to 3')	Comment
**ODA-01**	EcZ-A/S-RT-f	gttccattggttcaaacatagtttctctcc	RT-PCR, real-time PCR
**ODA-02**	EcZ-A/S-RT-r	cacatcttaacggtgaagctgaagtagaaa	RT-PCR, PEA
**ODA-03**	Ec-16S-rRNA-RT-f	tgaagaccggcggaagaag	RT-PCR, real-time PCR
**ODA-04**	Ec-16S-rRNA-RT-r	cactttatgaggtccgcttgct	RT-PCR, real-time PCR
**ODA-05**	EcftsZ-RT-f	tgaagaccggcggaagaag	RT-PCR, real-time PCR
**ODA-06**	EcftsZ-RT-r	gaagcaaatgcacggatggt	RT-PCR, real-time PCR
**ODA-07**	EcZ-A/S-PE2	ccaatggaacttaccaatgacgcgg	PEA
**ODA-08**	EcZ-A/S-RTr2	cctcaggcgacaggcacaaatcggagagaaact	PEA
**ODA-09**	pFPV-27-mutgfp-RT-f	tcggttatggtgttcaatgctt	Real-time PCR
**ODA-10**	pFPV27-mutgfp-RT-r	acttgacttcagcacgtgtcttg	Real-time PCR
**ODA-11**	EcftsA-RT-f	caaggcgacgcacagaaaac	Real-time PCR
**ODA-12**	EcftsA-RT-r	ttcaccccgcctttatcca	Real-time PCR
**ODA-13**	Ec-ftsZ-A/S-locus-f	ggggtacctgtctgcaccttccagcgcC	PCR
**ODA-14**	Ec-ftsZ-A/S-f	gggtaccttggtgataccgctaccgatttgaatcgtctg	Cloning
**ODA-15**	Ec-ftsZ-A/S-r	gctctagatttaacggattatgctcaggagccgtattattc ga	Cloning
**ODA-16**	EcZ-a/s-P1-f	gatcccgccgacgccgatgactttaatcaccgcgtcattgg taagttccattggttcaaacatagggtac	Cloning
**ODA-17**	EcZ-a/s-P1-r	cctatgtttgaaccaatggaacttaccaatgacgcggtgat taaagtcatcggcgtcggcgg	Cloning
**ODA-18**	EcZ-a/s-P2-f	gatccgtccaaccgctgttttacgcagcgcttgtgcatcgg tatttaccgcgaagaattcaacacggtac	Cloning
**ODA-19**	EcZ-a/s-P2-r	cgtgttgaattcttcgcggtaaataccgatgcacaagcgct gcgtaaaacagcggttggacg	Cloning
**ODA-20**	EcZ-a/s-P3-f	gatcccagccgcattgcggccaacttctggattagcgccag cgcccagtcctttggtgataccgcggtac	Cloning
**ODA-21**	EcZ-a/s-P3-r	cgcggtatcaccaaaggactgggcgctggcgctaatccaga agttggccgcaatgcggctgg	Cloning
**ODA-22**	EcZ-a/s-P1-10f	gatcccgccgacgccgatgactttaatcaccgcgtcattgg ccattggttcaaacatagggtac	Cloning
**ODA-23**	EcZ-a/s-P1-10r	cctatgtttgaaccaatggccaatgacgcggtgattaaagt catcggcgtcggcgg	Cloning
**ODA-24**	EcZ-a/s-P2-10f	gatccgtccaaccgctgttttacgcagcgcttgtgcatcgg ccgcgaagaattcaacacggtac	Cloning
**ODA-25**	EcZ-a/s-P2-10r	cgtgttgaattcttcgcggccgatgcacaagcgctgcgtaa aacagcggttggacg	Cloning
**ODA-26**	EcZ-a/s-P3-10f	gatcccagccgcattgcggccaacttctggattagcgccag cgcctttggtgataccgcggtac	Cloning
**ODA-27**	EcZ-a/s-P3-10r	cgcggtatcaccaaaggcgctggcgctaatccagaagttgg ccgcaatgcggctgg	Cloning
**ODA-28**	EcZ-a/s-P3-T-f	cgggatcccagccgcattgcggc	Cloning
**ODA-29**	EcZ-a/s-P1-T-r	ggggtaccctatgtttgaaccaatggaacttaccaatg	Cloning
**ODA-30**	3'Lig-Prim-compl-KS-r	cgaggtcgacggtaggtacccc	3’ RACE
**ODA-31**	3'Lig-Prim-KS-f	ggggtacctaccgtcgacctcg	3’ RACE

**ODA-32**	StfZ-ΔRBS-f	gatttgtgcctgtcgcc	Cloning
**ODA-33**	StfZ-ΔRBS-r	aaactatgtttgaaccaatggaac	Cloning
**ODA-34**	P1-mut-f	gcacaaatcggagagaaactatgtttgaaccaatggagctc accaatgacgcggtgattaaagtcatcggcgtcggcg	Cloning
**ODA-35**	P1-mut-r	cgccgacgccgatgactttaatcaccgcgtcattggtgagc tccattggttcaaacatagtttctctccgatttgtgc	Cloning
**ODA-36**	P2-mut-f	tgcgcgagcgcattgaaggtgttgaattcttcgcggtcaac accgatgcacaagcgctgcgtaaaacagcggttggac	Cloning
**ODA-37**	P2-mut-r	gtccaaccgctgttttacgcagcgcttgtgcatcggtgttg accgcgaagaattcaacaccttcaatgcgctcgcgca	Cloning
**ODA-38**	P3-mut-f	acagacgattcaaatcggtagcggtatcaccaaaggattgg gtgctggcgctaatccagaagttggccgcaatgcggct	Cloning
**ODA-39**	P3-mut-r	agccgcattgcggccaacttctggattagcgccagcaccca atcctttggtgataccgctaccgatttgaatcgtctgt	Cloning
**ODA-40**	cspA-f	aggcttcggcttcatcactc	Real-time PCR
**ODA-41**	cspA-r	ggttacgttaccagctgccg	Real-time PCR
**ODA-42**	rpoH-f	tcgccgttcactggatcaaa	Real-time PCR
**ODA-43**	rpoH-r	tgaagaacagtttgcgctgc	Real-time PCR
**ODA-44**	katG-f	ccaattgctgacggtttccg	Real-time PCR
**ODA-45**	katG-r	gtgaagacgccgtttttgct	Real-time PCR
**ODA-46**	StfZ-552-f	ggtgataccgctaccgatttg	Real-time PCR
**ODA-47**	StfZ-552-r	gcgcattgaaggtgttgaatt	Real-time PCR
**ODA-48**	StfZ-474-f	tcaacaccttcaatgcgctc	Real-time PCR
**ODA-49**	StfZ-474-r	ggaacttaccaatgacgcgg	Real-time PCR
**ODA-50**	StfZ-366-f	cccaccgccgtcgaataata	Real-time PCR
**ODA-51**	StfZ-366-r	ctcagcgcgtgtttcatacg	Real-time PCR
