# Supplementary figures and images for: Complete identity and expression of StfZ, the cis-antisense RNA to the mRNA of the cell division gene ftsZ, in Escherichia coli
Source: Front Microbiol. 2022 Oct 19;13:920117. doi: 10.3389/fmicb.2022.920117 (PMC9628754; doi:10.3389/fmicb.2022.920117)

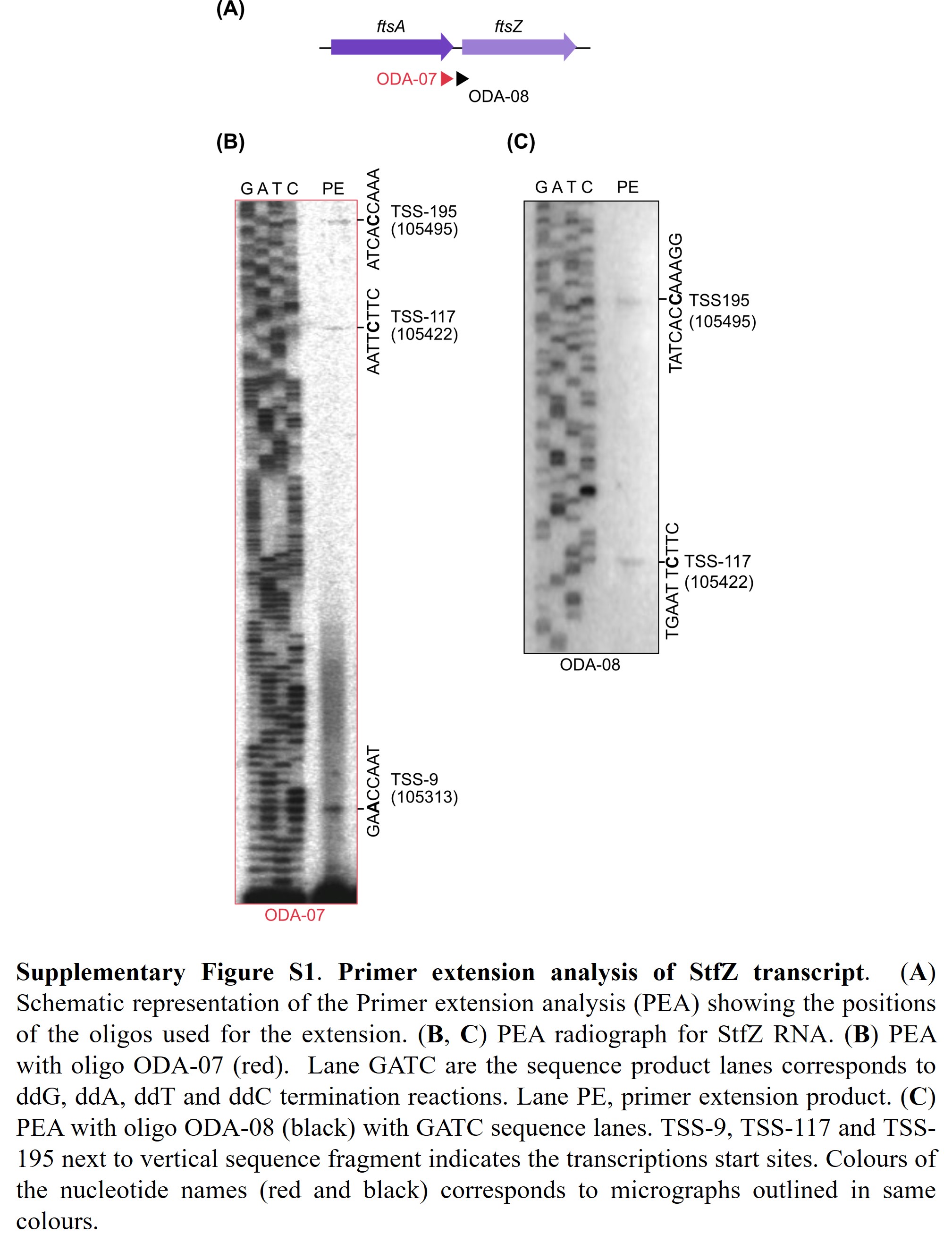

Supplement: Supplementary file 5 [file Image_1.JPEG]

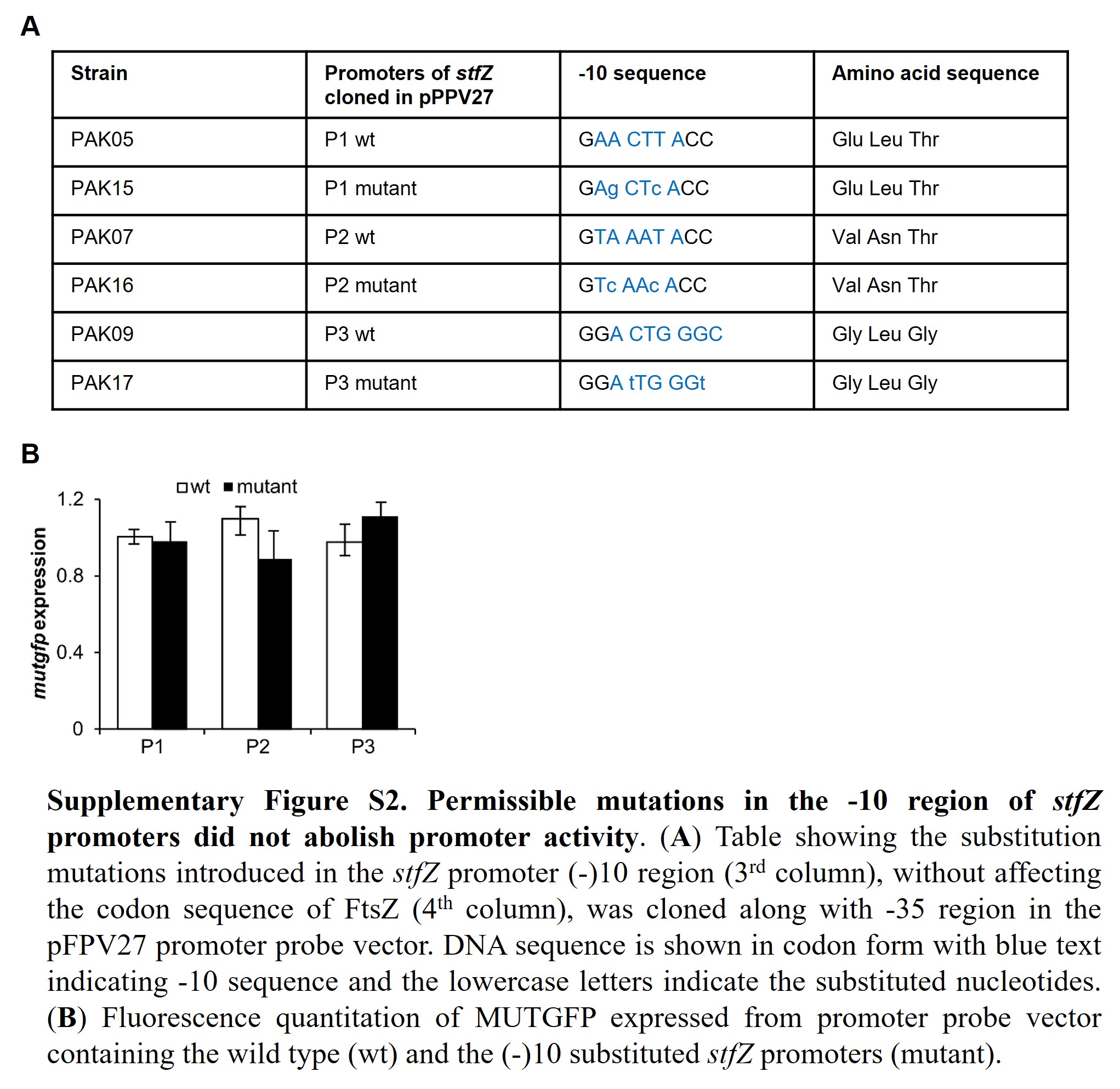

Supplement: Supplementary file 6 [file Image_2.JPEG]

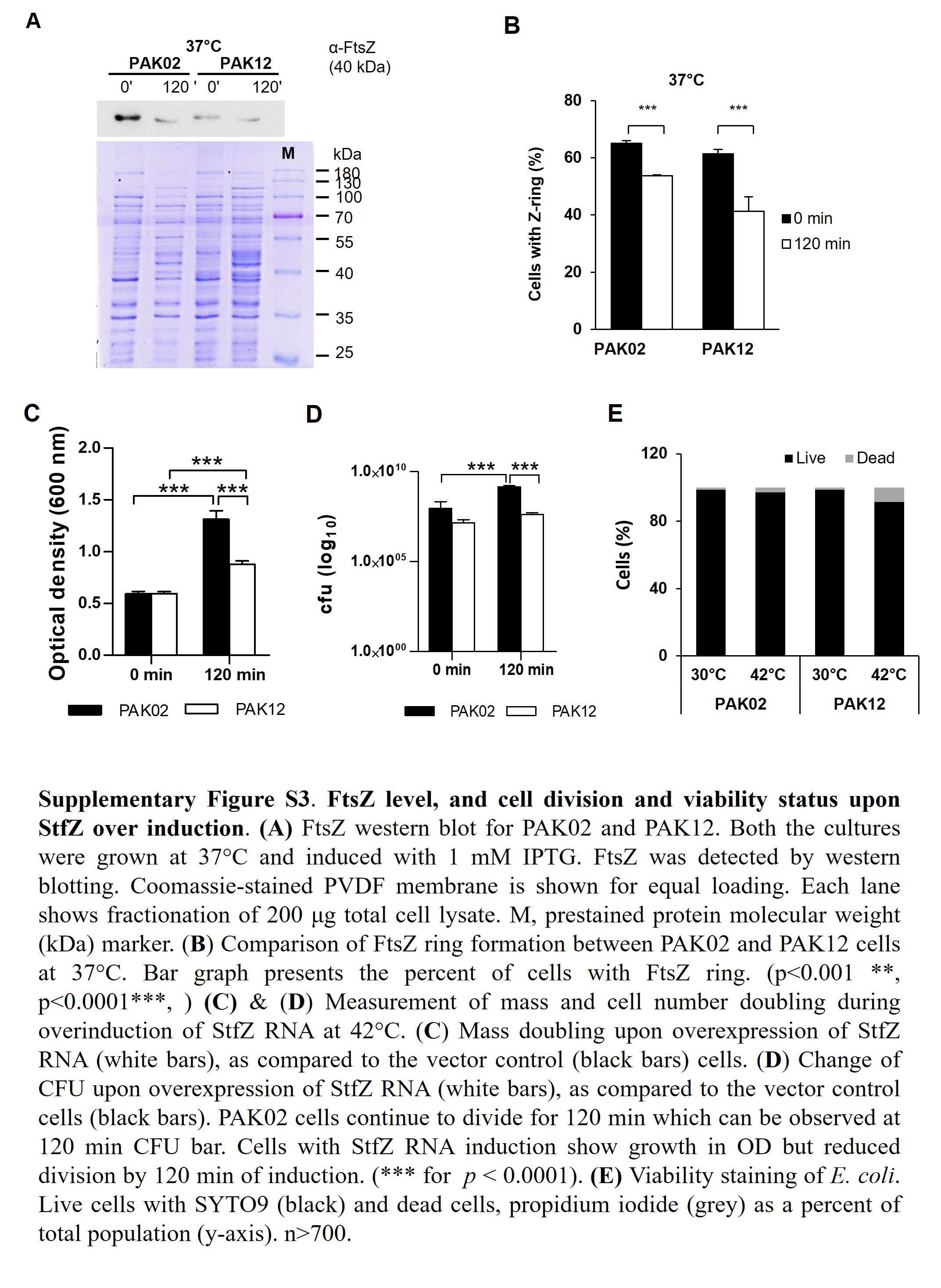

Supplement: Supplementary file 7 [file Image_3.JPEG]

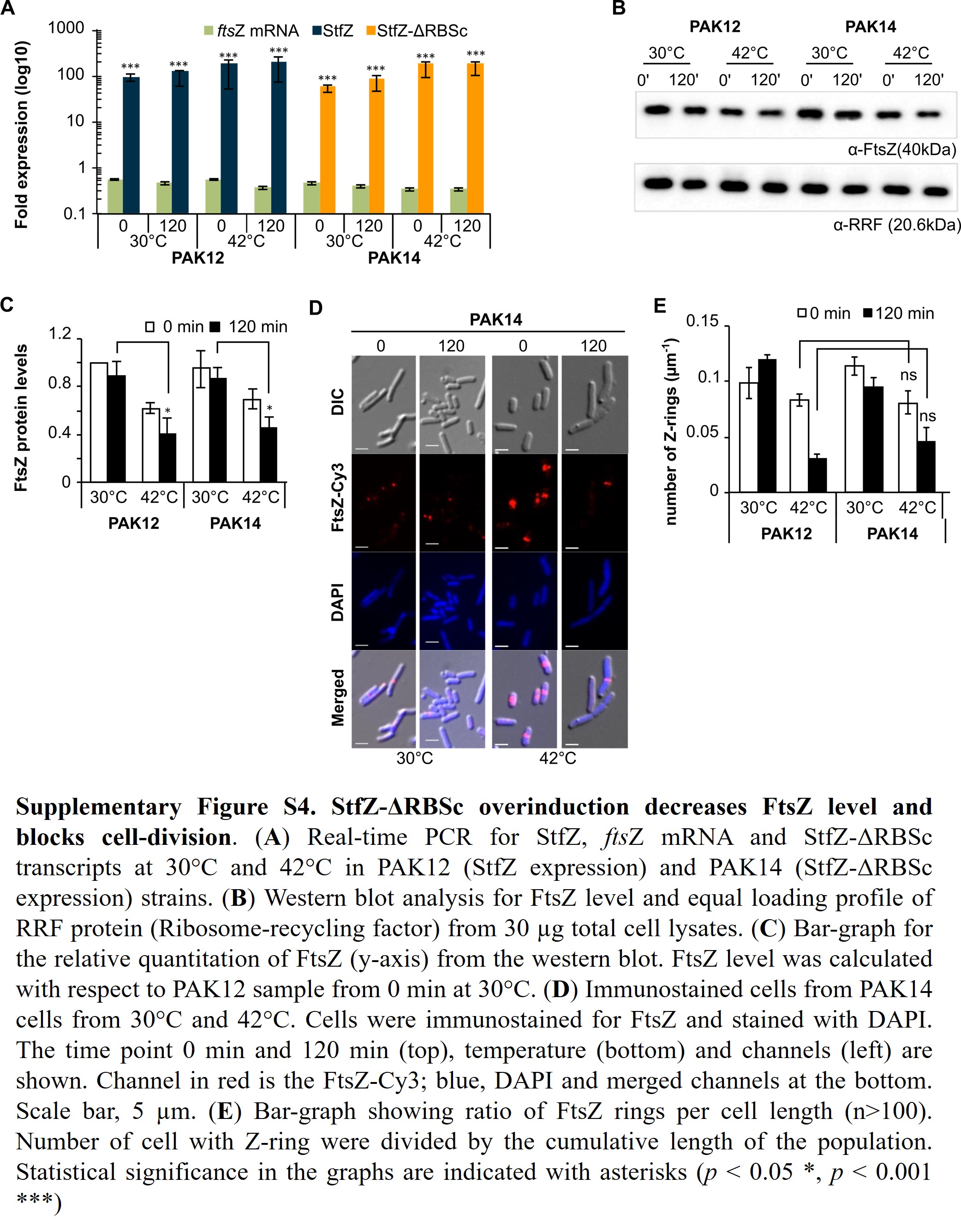

Supplement: Supplementary file 8 [file Image_4.JPEG]
